# Intra-Articular Injection of Umbilical Cord Mesenchymal Stem Cells Loaded With Graphene Oxide Granular Lubrication Ameliorates Inflammatory Responses and Osteoporosis of the Subchondral Bone in Rabbits of Modified Papain-Induced Osteoarthritis

**DOI:** 10.3389/fendo.2021.822294

**Published:** 2022-01-14

**Authors:** Aifeng Liu, Jixin Chen, Juntao Zhang, Chao Zhang, Qinxin Zhou, Puyu Niu, Ye Yuan

**Affiliations:** ^1^ Department of Orthopaedic Surgery, First Teaching Hospital of Tianjin University of Traditional Chinese Medicine, Tianjin, China; ^2^ National Clinical Research Center for Chinese Medicine Acupuncture and Moxibustion, Tianjin, China; ^3^ Tianjin Key Laboratory of Materials Laminating Fabrication and Interface Control Technology, Hebei University of Technology, Tianjin, China

**Keywords:** graphene oxide, umbilical cord mesenchymal stem cells, knee osteoarthritis, cartilage repair, osteoporosis

## Abstract

**Aim:**

This study is to investigate the effects of umbilical cord mesenchymal stem cells (UCMSCs) loaded with the graphene oxide (GO) granular lubrication on ameliorating inflammatory responses and osteoporosis of the subchondral bone in knee osteoarthritis (KOA) animal models.

**Methods:**

The KOA animal models were established using modified papain joint injection. 24 male New Zealand rabbits were classified into the blank control group, GO group, UCMSCs group, and GO + UCMSCs group, respectively. The concentration in serum and articular fluid nitric oxide (NO), interleukin-6 (IL-6), tumor necrosis factor-α (TNF-α), type II collagen (COL-II), and glycosaminoglycan (GAG) was detected using ELISA, followed by the dissection of femoral condyles and staining of HE and Micro-CT for observation *via* the microscope.

**Results:**

GO granular lubrication and UCMSCs repaired the KOA animal models. NO, IL-6, TNF-α, GAG, and COL-II showed optimal improvement performance in the GO + UCMSCs group, with statistical significance in contrast to the blank group (*P <*0.01). Whereas, there was a great difference in levels of inflammatory factors in serum and joint fluid. Micro-CT scan results revealed the greatest efficacy of the GO + UCMSCs group in improving joint surface damage and subchondral bone osteoporosis. HE staining pathology for femoral condyles revealed that the cartilage repair effect in GO + UCMSCs, UCMSCs, GO, and blank groups were graded down.

**Conclusion:**

UCMSCs loaded with graphene oxide granular lubrication can promote the secretion of chondrocytes, reduce the level of joint inflammation, ameliorate osteoporosis of the subchondral bone, and facilitate cartilage repair.

## Introduction

Knee osteoarthritis (KOA) is a degenerative bone and joint disease and most prevalent in the middle-aged and elderly (1). Besides, it is characterized by joint pain and limited range of motion in the knee and the osteoporosis of the subchondral bone, with progressive intra-articular cartilage and narrowing of the joint cavity (2–6). By 2032, the proportion of people aged 45 and over with a diagnosis of knee osteoarthritis by a doctor is estimated to increase from 13.8% to 15.7% (7). With the effect of aging and increasing obesity, KOA is expected to be the single biggest reason for disability by 2030. Apart from that, it has become a major public health issue along with the increasing morbidity of KOA (8–10). Currently, major non-pharmacological treatments such as physical therapy, medical education and self-management, weight loss, non-steroidal anti-inflammatory drugs, and intra-articular injections are recommended in the guidelines. Unfortunately, these treatments only relieve pain and fail to slow down cartilage degeneration and promote cartilage repair (11).

In recent years, many researchers have begun to focus on the influence of the subchondral bone on the occurrence of KOA (12–14). The cartilage and subchondral bones are closely linked in the mechanical and biochemical microenvironment and are considered to be the key structural unit for maintaining the stability of the knee joint (15, 16). When cartilage is damaged, it is difficult to regenerate the articular cartilage due to insufficient vascularization. Besides, when the cartilage is destroyed and the subchondral bone is exposed, there is an imbalance in the otherwise normal bone and cartilage metabolism, and the balance between osteogenesis and osteolysis is broken, thus disrupting the normal remodeling process of the subchondral bone. In addition, this remodeling process inevitably leads to changes in the microstructure of the subchondral bone, especially changes in the vasculature of the subchondral bone, causing abnormalities in the normal interaction channels between the cartilage and subchondral bone, which in turn further results in an acceleration of the bone remodeling process that is the rapid development of OA to advanced stages, bringing about a vicious circle (17, 18). Other than that, subchondral bone provides nutrition for articular cartilage and influences articular cartilage metabolism (19). Therefore, inhibiting bone loss from subchondral bone and providing nutrition for articular cartilage may be a potential target for KOA treatment (2, 20). Actually, total knee arthroplasty remains the final treatment option for patients with end-stage KOA (21). However, there are many complications after TKA, such as loosening of the prosthesis, the infection, the thromboembolic disease, and poor wound healing. Considering that, many patients wish to treat KOA through cartilage repair instead of TKA. In recent years, mesenchymal stem cells (MSCs) have been regarded as a potentially more promising source of cellular therapy for KOA (22–24). Due to the enormous osteogenic potential, minimally-invasive injections, and immunomodulatory biological properties of MSCs, numerous researchers have demonstrated the effects of MSCs for ameliorating joint surface damage and osteoporosis of the subchondral bone in KOA (25–27). However, the low survival rate of cells during transplantation and injection, suboptimal cell potency, and the most suitable stem cell type and dose are controversial, resulting in a failure to achieve the expected improvement in knee function and cartilage repair (28). To overcome these limitations, bioactive scaffold materials as an adjunct to mesenchymal stem cell therapy for KOA attract increasing attention (29–31).

Graphene oxide (GO) has aroused considerable interest in tissue engineering and regenerative medicine due to its specific characteristics, such as physiochemical, antibacterial, and biological capabilities (32, 33). Owing to its larger surface area and electrostatic adsorption capacity, GO can aggregate UCMSCs and anchor them in the damaged tissue. Previous studies have found that GO particle lubricants can promote the repair of KOA chondrocytes through lubrication (34). In addition, GO can facilitate stem cell differentiation for cartilage repair and ameliorate osteoporosis of the subchondral bone (35, 36). Hence, UCMSCs loaded with GO granular lubrication as new tissue engineering materials provide new ideas and approaches for the treatment of KOA.

There are few studies on the combined applications of UCMSCs loaded with GO granular lubrication for the treatment of KOA to assessing cartilage repair and osteoporosis of the subchondral bone comprehensively. Thus, twenty-four New Zealand rabbits’ KOA models were induced by modified papain joint injection in this study. Among them, 18 are treated by joint cavity injection of UCMSCs and GO granular lubrication so as to observe the effect of serum and joint fluid inflammatory factor levels, ameliorate osteoporosis of the subchondral bone, and assess cartilage repair macroscopically, histologically. 

## Materials and Methods

### Experimental Equipment

UCMSCs were provided by Tianjin Boya Stem Cell Technology Co., Ltd. Graphene oxide particles were provided by the Hebei University of Technology. Sodium Hyaluronate Injection (Japan, Seikagaku Corporation), NO Kits (USA, Cranford, CCC company), IL-6 kits (USA, Cranford, CCC company), TNF-α kits (USA, Cranford, CCC company), GAG kits (USA, Cranford, CCC company), COL-II kits (USA, Cranford, CCC company), micro-CT (Germany, Siemens Company), centrifuge (USA, Thermo Fisher Scientific Company), and Microplate Reader (USA, Thermo Fisher Scientific) were also applied.

24 male New Zealand white rabbits (12 weeks in age; 2 kg in weight) were chosen as the experimental subjects. The experimental protocols based on an animal model were approved by the animal experimental center at Tianjin University of Traditional Chinese Medicine.

### UCMSCs Cultured

The UCMSCs used in the experiment were purchased from Tianjin Boya Stem Cell Technology Co., Ltd, and then they were isolated, cultured, and tested in Boya Stem Cell Technology laboratory. After that, the isolated P5 generation UCMSCs were transferred to DMEM containing 10% FBS and 1% penicillin-streptomycin.

### GO Granular Lubrication

GO granules were bought from Hebei University of Technology. After mixing 15 µg/ml of GO granules with the Sodium Hyaluronate Injection, the GO granular lubrication is disinfected with UV light for 1 hour and re-encapsulated (37).

### Modified Papain Release Agent Model

**Figure 1:**
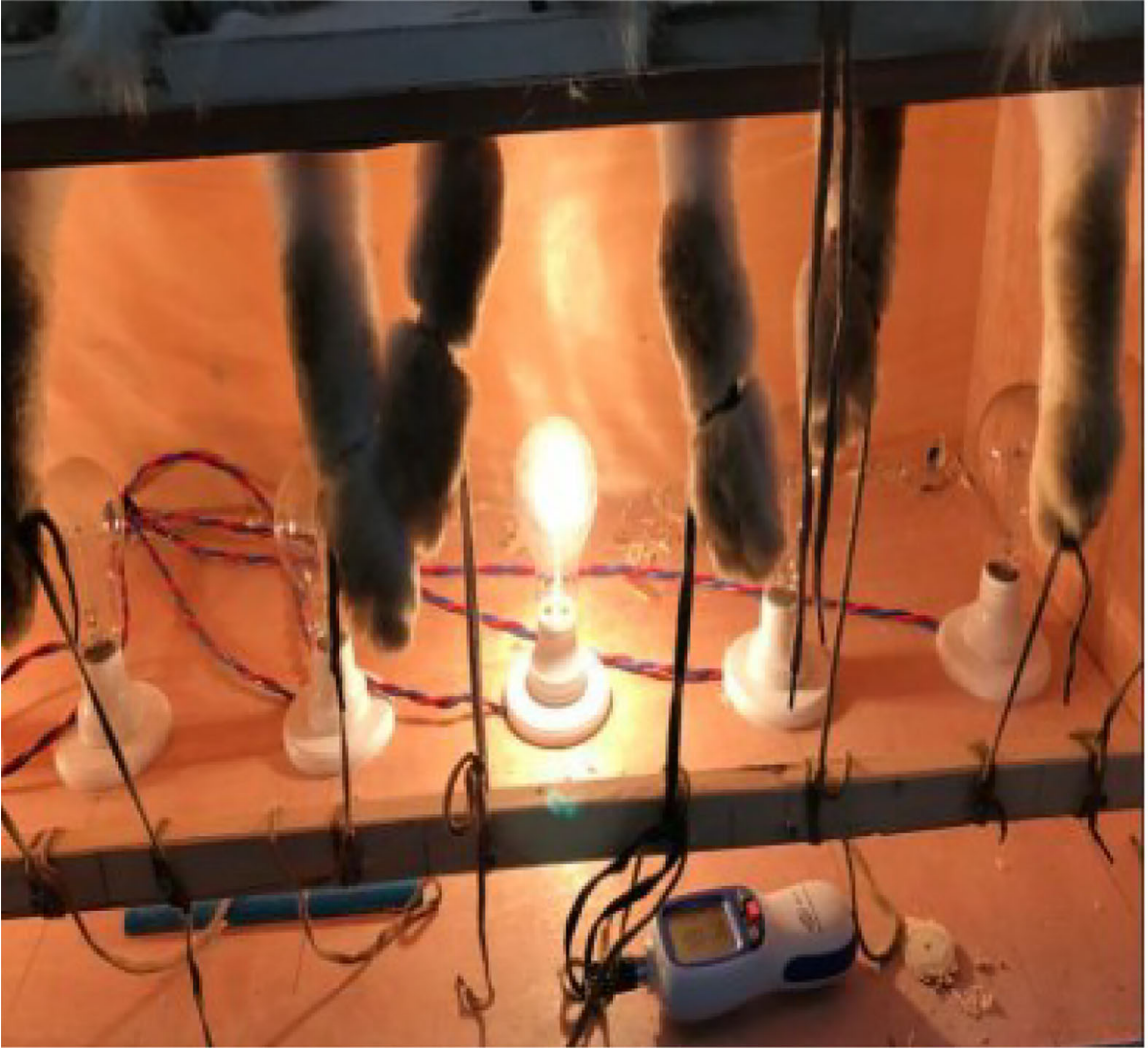
Establishment of modified papain release agent model.

After the review by the animal experiment center at Tianjin University of Traditional Chinese Medicine, the animal experiment scheme and associated materials included by this project satisfied the ethical demands on experimental animal welfare and animal experiment. The weighed animals received anesthetization treatment in chloral hydrate solution (3 ml/kg) at 35% concentration. Paroxamer at 35% concentration was loaded with 4% papain as the drug carrier. A temperature-sensitive *in situ* gel-controlled release injection was prepared, and the injection dose standard was 0.05 ml (1.6 U)/joint. The injection was administered to the rabbit from the medial or lateral space of the right knee joint, and the bloodless fluid was withdrawn, followed by the papain mixture being injected into the knee joint cavity. After the injection, both knee joints and the following parts of the rabbits were placed in a self-heated chamber featured with the temperature of 50°C for 2 h. During the heating period, it should be ensured that the chamber was heated for 2 h, and the temperature of the thermometer inside the chamber was kept at 50°C, while the parts above the knee joints were not affected by the heat (**Figure 1**). After 2 h, the rabbits were taken out and allowed to move freely and eat and drink normally. After 2 h, the rabbits were released and free movement was allowed, with normal provision of water and food. Following the construction of the KOA animal model, four-week observation was performed, survival and activity included. The rabbits were executed using ear vein air embolization upon the completion of the observation. Afterwards, the right knee joint cavity was opened for resection, followed by the removal of excess muscle and ligament aseptically. The variations of cartilage and synovium were detected and documented. Based on the preclinical small-animal PET/SPECT/CT system, a micro-CT scan was conducted on the right knee specimens at a thickness of 80 μm under the animal model.

### Detection of the KOA Rabbit Model

**Figure 2:**
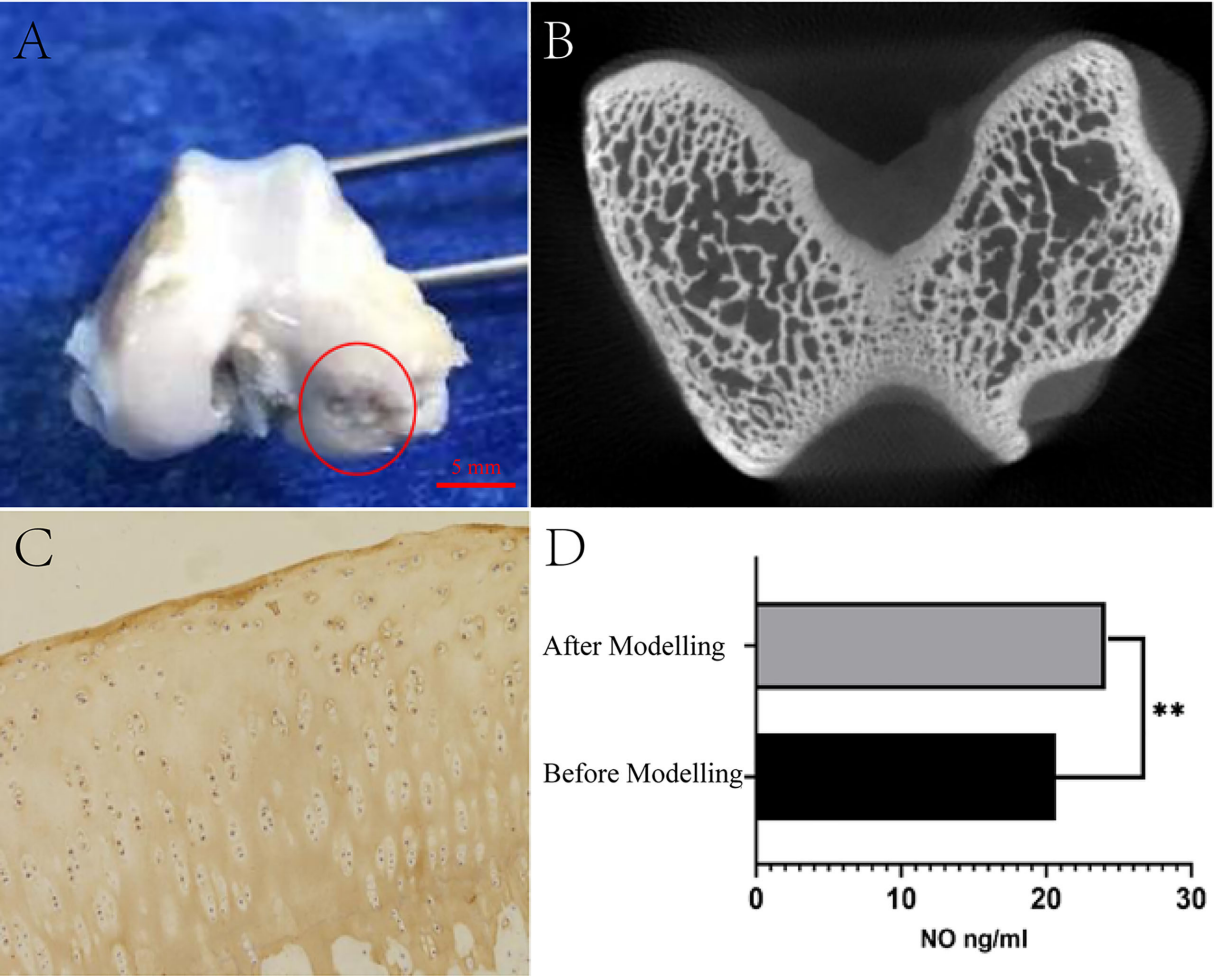
Test of KOA model generated by modified papain joint injection. **(A)** Morphological observation of cartilage. **(B)** Micro-CT scan results of articular cartilage and subchondral bone. **(C)** Staining results of type II collagen. **(D)** Serum NO detection results (**P <0.01 as compared to the blank group, n=3).

After the KOA animal model was established, daily activity, survival rate, and adverse effects were observed. After 4 weeks, the rabbits were executed by air embolization of the auricular vein. The right knee joint was amputated, followed by the removal of excess muscle and ligament in aseptic surgery. The variations of cartilage and synovium were detected and documented (**Figure 2A**).

By adopting a preclinical small animal PET/SPECT/CT system with a tomographic thickness of 80 μm, Micro-CT scans were performed on right knee specimens from experimental rabbits to assess damage to the articular cartilage and subchondral bone (**Figure 2B**).

The specimens were stored in 10% formalin solution and decalcified using a decalcifier for 28 d. As for the decalcification solution, it was concentrated in 10%, embedded in paraffin, sectioned (5 μm), immunohistochemically stained for type II collagen, and then observed using microscopy for examining the chondrocyte morphology and distribution (**Figure 2C**).

Before and after modeling, 5ml of venous blood was drawn from the ear margin of the KOA animal model under aseptic conditions, injected into a heparinized centrifuge tube, centrifuged at 3000 r/min for 10 minutes to collect the serum and stored at -20°C. Besides, the serum NO concentration was determined by ELISA using the NO kit (**Figure 2D**). 

### Experimental Grouping

The 24 animal models were classified into the blank control group with six rabbits and the treatment experimental group with 18 rabbits. Specifically, the experimental group could be subdivided into GO granule lubrication group (GO group), UCMSCs group (UCMSCs group), as well as UCMSCs loaded with GO granule lubrication group (GO + UCMSCs group).

### Therapeutic Method

The GO granule lubrication concentration was 15 μg/ml GO + 0.25% HA. The UCMSCs concentration was 5 ×10^6^ cells/ml. Injection was conducted on all joints once, with a treatment cycle of 28 days. GO group: 0.5 ml of the GO granule lubrication was injected into the right knee cavity under the animal model. UCMSCs group: 0.5 ml of the UCMSCs was injected into the lumen of the right knee cavity under the animal model. GO + UCMSCs group: 0.5 ml of the UCMSCs loaded with GO granule lubrication was injected into the right knee joint cavity under the animal model (34, 37).

### Observation Index

After the eight-week treatment, 5 ml of auricular venous blood was retrieved from two experimental KOA animal models aseptically, followed by injection into heparinized centrifugation tubes. The serum received centrifugation at 3000 r/min in 10 min, and kept at -20°C. 1 ml of joint fluid was extracted from the knee cavity under the animal model. The articular fluid was gathered, centrifuged at 5000 r/min in 10 min and kept at -20°C. NO, IL-6, TNF-α, GAG, and Col-II concentrations of serum and articular fluid were observed using ELISA.

The animals were executed by air embolization, and the right knee specimens were scanned by Micro-CT with a tomographic thickness of 80 μm to assess the damage to the articular cartilage and subchondral bone. Then, the areas of cartilage degeneration defects were observed and subjected to HE staining for pathology.

### Statistical Methods

SPSS 25.0 software was employed for statistical analysis. The measurement data were represented by ANOVA for multi-group comparison and the LSD-T test for further pale comparison, when α=0.05. 

## Results

### NO Results

**Figure 3:**
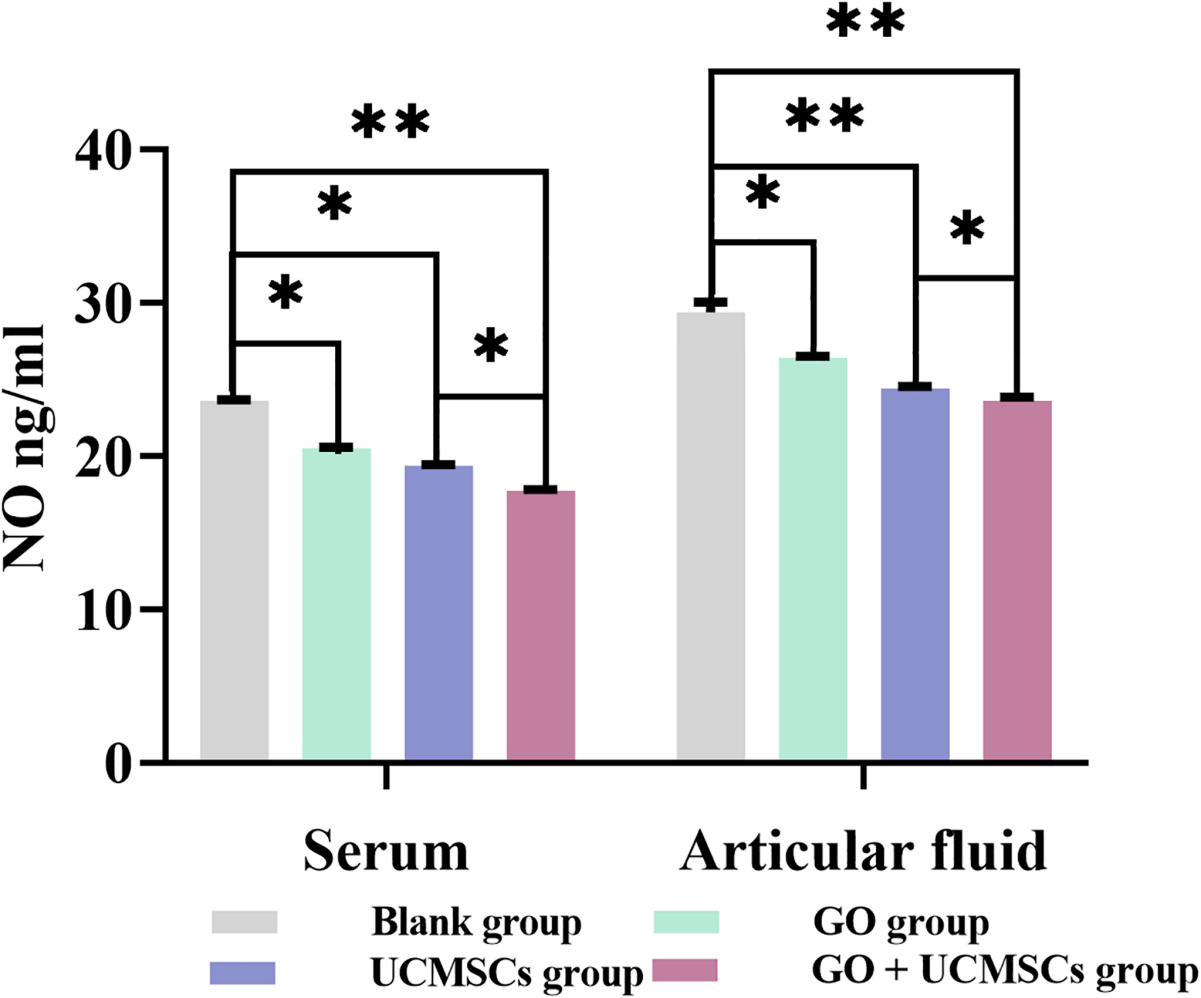
The NO results of serum and articular fluid after eight weeks of treatment from blank, GO, UCMSCs, GO + UCMSCs groups (n=6). The bars represent the mean and standard deviation of data. Statistical significance between groups is shown by: *P < 0.05, **P < 0.01.

The NO results of serum and articular fluid were shown in **Table 1** and **Figure 3**. The GO + UCMSCs group had a lower mean value of serum NO compared with the blank group. The difference showed high statistical significance (*P* < 0.01). The UCMSCs group and the GO group had lower mean values of serum NO compared with the blank group (*P* < 0.05). The GO + UCMSCs group had a lower mean value of serum NO compared with the UCMSCs group (*P* < 0.05).

**Table 1:** Serum and articular fluid NO results after treatment ( $\bar{x}$ ± s, ng/ml).

Group	Serum	Articular fluid	*P*
Blank group	23.662 ± 0.056	29.431 ± 0.667	0.000
GO group	20.544 ± 0.085^1^	26.457 ± 0.101^4^	0.020
UCMSCs group	19.424 ± 0.046^2^	24.452 ± 0.135^5^	0.012
GO + UCMSCs group	17.799 ± 0.049^3^	23.658 ± 0.210^6^	0.038

^1 – 6^Compared with the Blank group respectively, P values were 0.032, 0.011, and 0.000; 0.020, 0.005, and 0.003. P values for serum and articular fluid in the GO + UCMSC group compared to the UCMSC group were 0.012 and 0.032 respectively.

The UCMSCs group and the GO + UCMSCs group had lower mean values of articular fluid NO compared with the blank group (*P* < 0.01). The GO group had a lower mean value of articular fluid NO compared with the blank group (*P* < 0.05). The GO + UCMSCs group had a lower mean value of articular fluid NO compared with the UCMSCs group (*P* < 0.05).

According to the NO results of serum and articular fluid, there was a significant statistical difference among groups (*P* < 0.05).

### IL-6 Results

**Figure 4:**
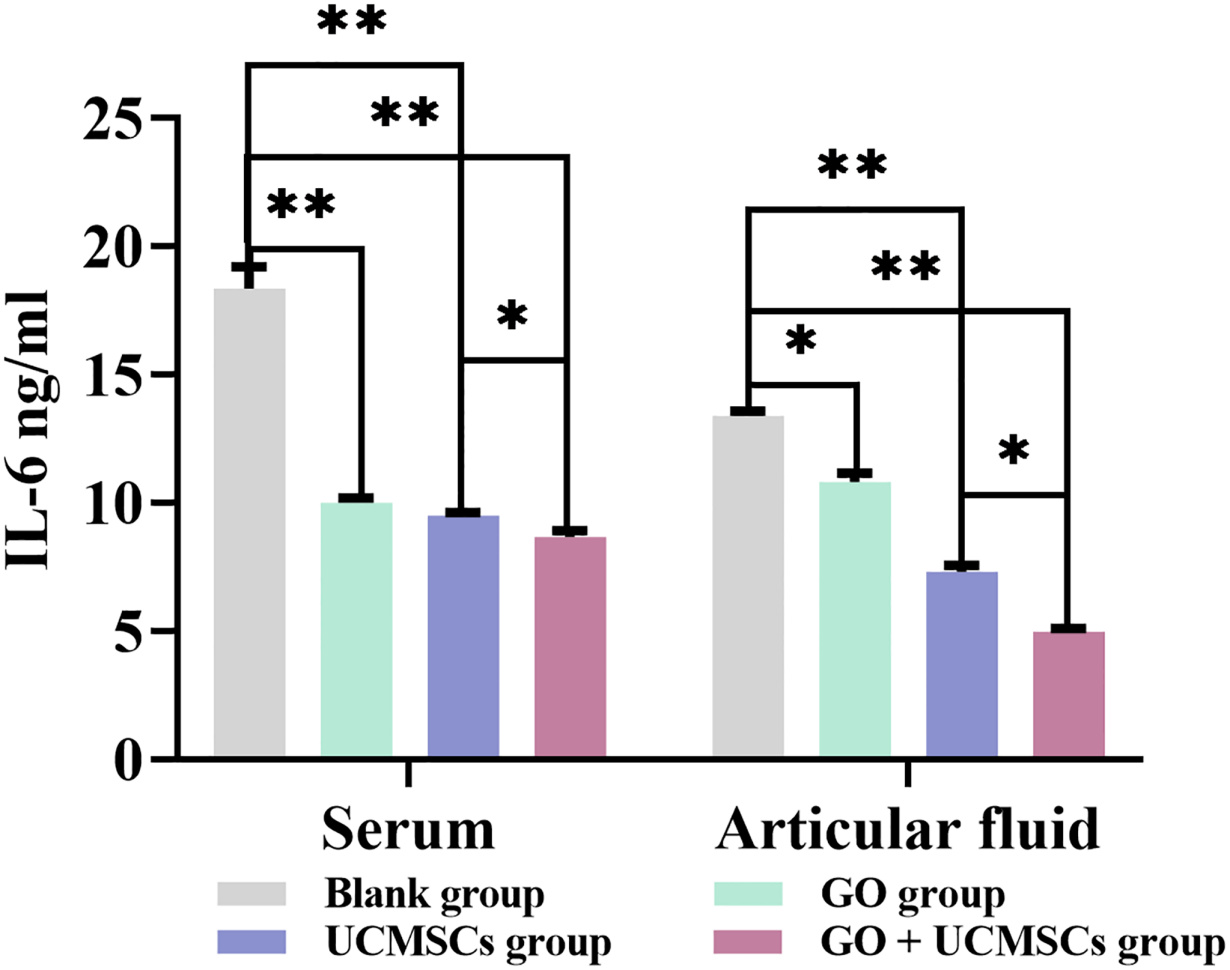
The IL-6 results of serum and articular fluid after eight weeks of treatment from blank, GO, UCMSCs, GO + UCMSCs groups (n=6). The bars represent the mean and standard deviation of data. Statistical significance between groups is shown by: *P < 0.05, **P < 0.01.

The IL-6 results of serum and articular fluid were shown in **Table 2** and **Figure 4**. The GO + UCMSCs group, the UCMSCs group, and the GO group had lower mean values of serum IL-6 compared with the blank group (*P* < 0.01). The GO + UCMSCs group had a lower mean value of serum IL-6 compared with the UCMSCs group (*P* < 0.05).

**Table 2:** Serum and articular fluid IL-6 results after treatment ( $\bar{x}$ ± s, ng/ml).

Group	Serum	Articular fluid	*P*
Blank group	18.367 ± 0.861	13.407 ± 0.181	0.000
GO group	10.002 ± 0.191^1^	10.827 ± 0.353^4^	0.045
UCMSCs group	9.506 ± 0.123^2^	7.318 ± 0.254^5^	0.026
GO + UCMSCs group	8.680 ± 0.242^3^	4.990 ± 0.115^6^	0.010

^1 – 6^Compared with the Blank group respectively, P values were 0.003, 0.002, and 0.002; 0.045, 0.004, and 0.000. P values for serum and articular fluid in the GO + UCMSC group compared to the UCMSC group were 0.016 and 0.025 respectively.

The UCMSCs group and the GO + UCMSCs group had lower mean values of articular fluid IL-6 compared with the blank group (*P* < 0.01). The GO group had a lower mean value of articular fluid IL-6 compared with the blank group (*P* < 0.05). The GO + UCMSCs group had a lower mean value of articular fluid IL-6 compared with the UCMSCs group (*P* < 0.05).

According to the IL-6 results of serum and articular fluid, there were significant statistical differences among the GO group, the UCMSCs group, and the GO + UCMSCs group (*P* < 0.05) and a significant statistical difference in the blank group (*P* < 0.01). 

### TNF-α Results

**Figure 5:**
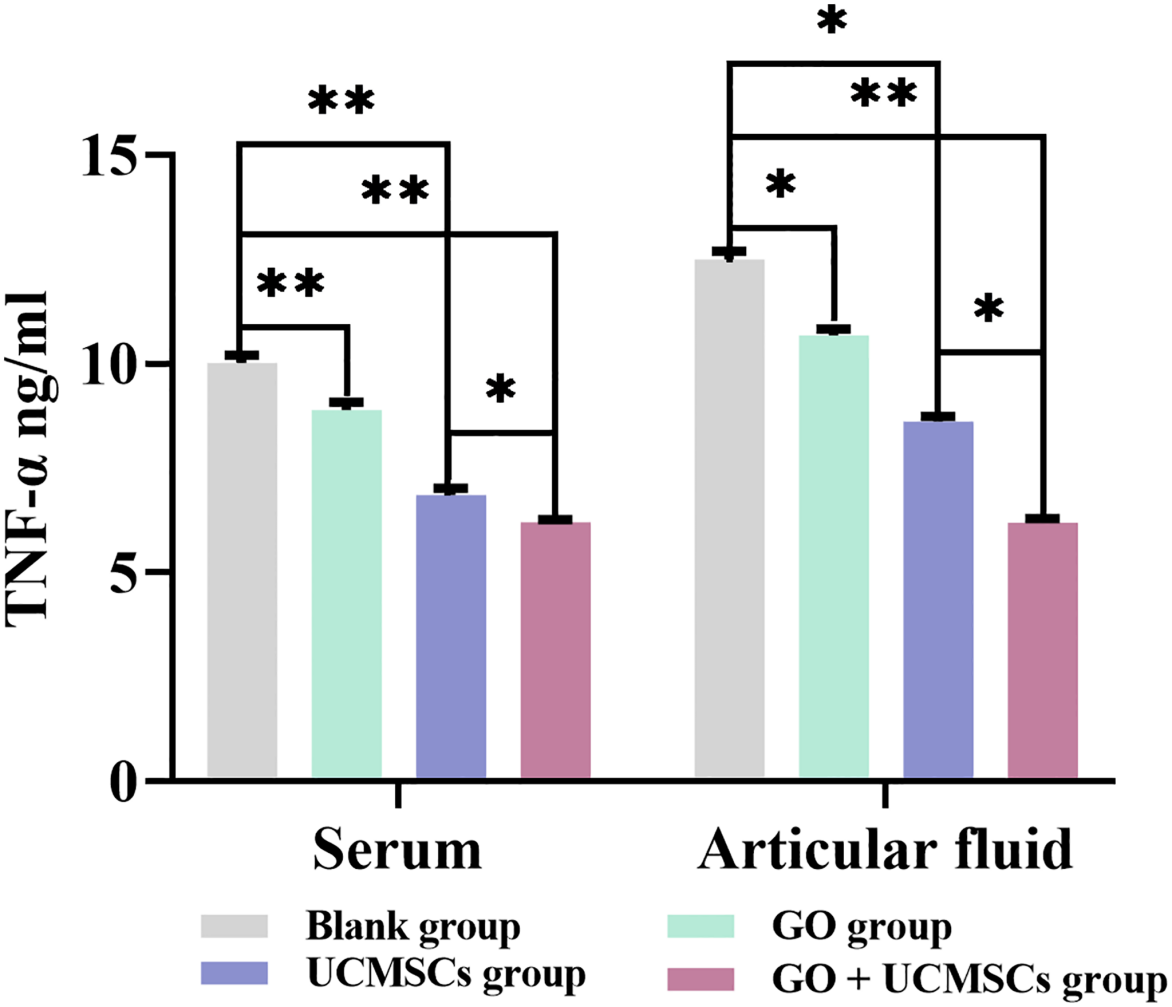
The TNF-α results of serum and articular fluid after eight weeks of treatment from blank, GO, UCMSCs, GO + UCMSCs groups (n=6). The bars represent the mean and standard deviation of data. Statistical significance between groups is shown by: *P < 0.05, **P < 0.01.

The TNF-α results of serum and articular fluid were shown in **Table 3** and **Figure 5**. The GO + UCMSCs group, the UCMSCs group, and the GO group had lower mean values of serum TNF-α compared with the blank group (*P* < 0.01). The GO + UCMSCs group had a lower mean value of serum TNF-α compared with the UCMSCs group (*P* < 0.05).

**Table 3:** Serum and articular fluid TNF-α results after treatment ( $\bar{x}$ ± s, ng/ml).

Group	Serum	Articular fluid	*P*
Blank group	10.013 ± 0.197	12.489 ± 0.201	0.000
GO group	8.891 ± 0.188^1^	10.676 ± 0.153^4^	0.045
UCMSCs group	6.856 ± 0.160^2^	8.613 ± 0.136^5^	0.037
GO + UCMSCs group	6.210 ± 0.058^3^	6.190 ± 0.105^6^	0.137

^1 – 6^Compared with the Blank group respectively, P values were 0.033, 0.002, and 0.000; 0.041, 0.013, and 0.001. P values for serum and articular fluid in the GO + UCMSCs group compared to the UCMSCs group were 0.142 and 0.005 respectively.

The GO + UCMSCs group had a lower mean value of articular fluid TNF-α compared with the blank group (*P* < 0.01). The UCMSCs group had a lower mean value of articular fluid TNF-α compared with the blank group (*P* < 0.05). The GO + UCMSCs group had a lower mean value of articular fluid TNF-α compared with the UCMSCs group (*P* < 0.05).

According to the TNF-α results of serum and articular fluid, statistically significant differences existed between the GO group and the UCMSCs group (*P* < 0.05), highly significant statistical difference existed in the blank group (*P* < 0.01), and no significant statistical difference existed in the GO + UCMSCs group (*P* > 0.05). 

### COL-II Results

**Figure 6:**
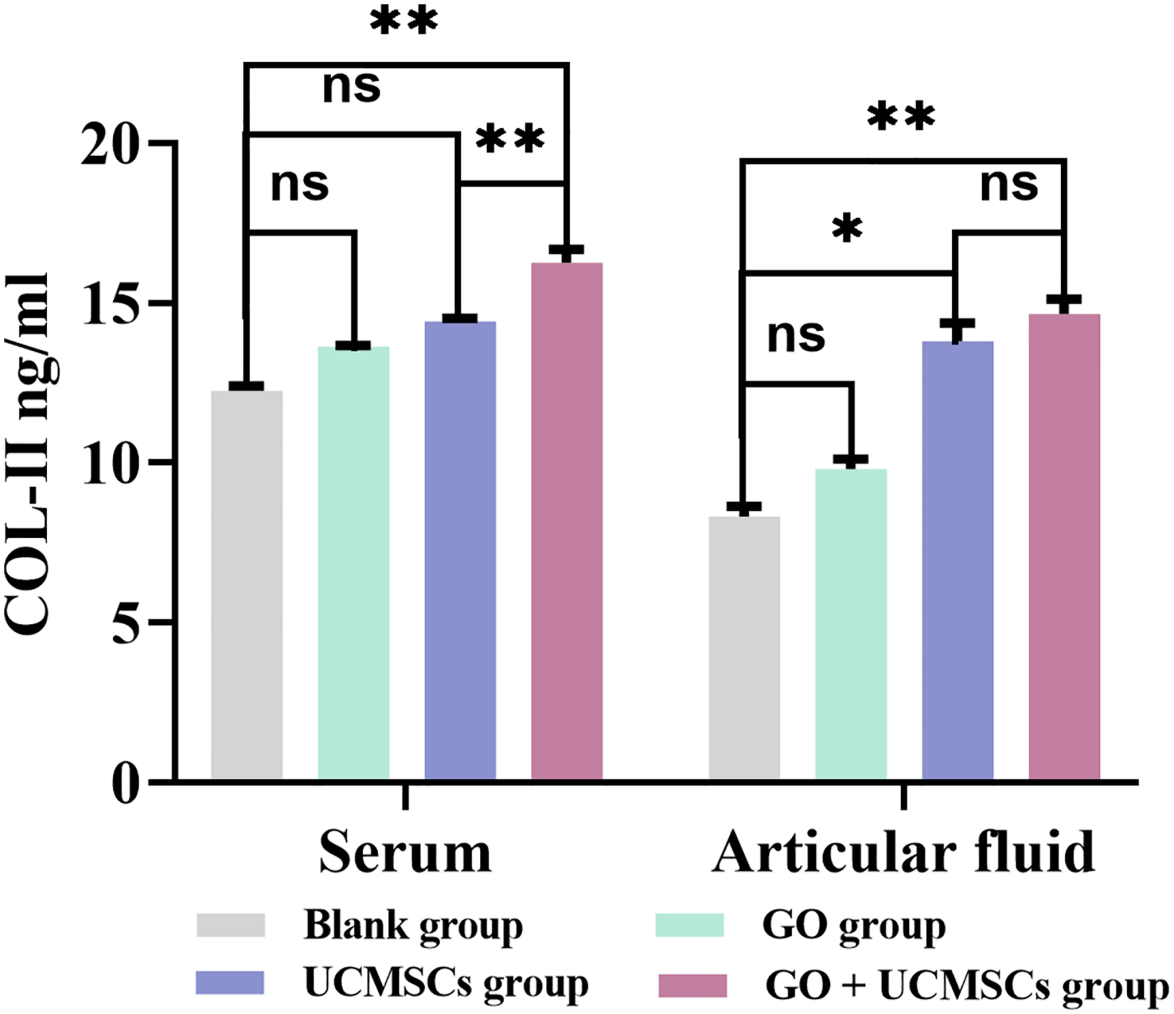
The COL-II results of serum and articular fluid after eight weeks of treatment from blank, GO, UCMSCs, GO + UCMSCs groups (n=6). The bars represent the mean and standard deviation of data. Statistical significance between groups is shown by: *P < 0.05, **P < 0.01, ns, not statistically significant (P ≥ 0.05).

The COL-II results of serum and articular fluid were shown in **Table 4** and **Figure 6**. The GO + UCMSCs group had a higher mean value of serum COL-II compared with the blank group (*P* < 0.01). The GO + UCMSCs group had a higher mean value of serum COL-II compared with the UCMSCs group (*P* < 0.01). There was no significant statistical difference in the GO group, the UCMSCs group, and the blank group *(P* > 0.05).

**Table 4:** Serum and articular fluid COL-II results after treatment ( $\bar{x}$ ± s, ng/ml).

Group	Serum	Articular fluid	*P*
Blank group	12.253 ± 0.147	8.328 ± 0.321	0.053
GO group	13.644 ± 0.028^1^	9.800 ± 0.331^4^	0.013
UCMSCs group	14.429 ± 0.092^2^	13.812 ± 0.569^5^	0.032
GO + UCMSCs group	16.257 ± 0.416^3^	14.655 ± 0.467^6^	0.109

^1 – 6^Compared with the Blank group respectively, P values were 0.381, 0.204, and 0.006; 0.085, 0.013, and 0.007. P values for serum and articular fluid in the GO + UCMSCs group compared to the UCMSCs group were 0.005 and 0.371 respectively.

The GO + UCMSCs group had a mean value of articular fluid COL-II compared with the blank group (*P* < 0.01). The UCMSCs group had a higher mean value of articular fluid COL-II compared with the blank group (*P* < 0.05). There was no significant statistical difference between the GO group and the blank group (*P* > 0.05). In the comparison between the GO + UCMSCs group and the UCMSCs group, no significant statistical difference was found in mean (*P* > 0.05).

According to the COL-II results of serum and articular fluid, significant statistical differences were found between the GO group and the GO + UCMSCs group (*P* < 0.05) while no significant statistical difference was found between the UCMSCs group and the blank group (*P *> 0.05).

### GAG Results

**Figure 7:**
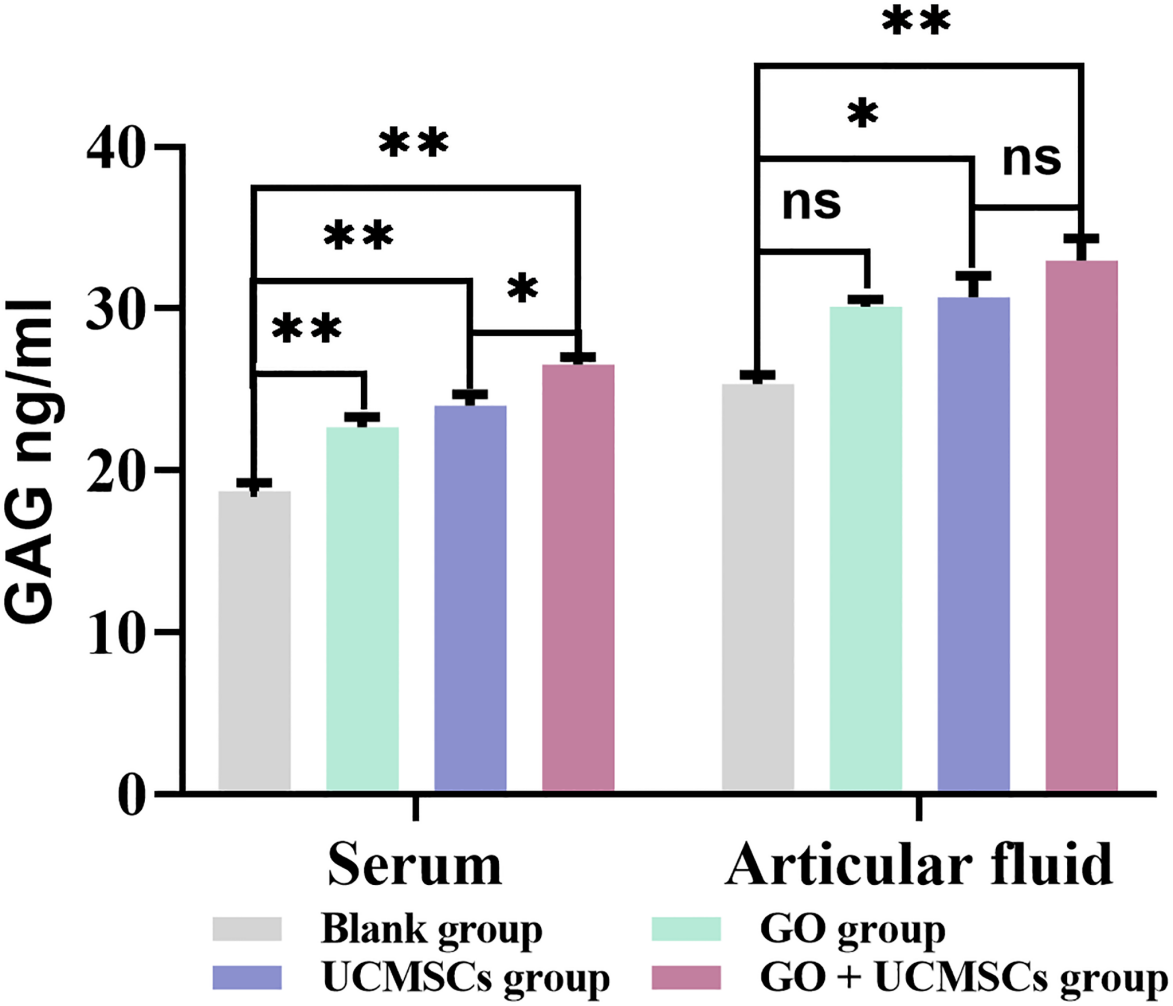
The GAG results of serum and articular fluid after eight weeks of treatment from blank, GO, UCMSCs, GO + UCMSCs groups (n=6). The bars represent the mean and standard deviation of data. Statistical significance between groups is shown by: *P < 0.05, **P < 0.01, ns, not statistically significant (P ≥ 0.05).

The results of GAG in serum and articular fluid were presented in **Table 5** and **Figure 7**. The mean values of serum GAG in the GO group, the UCMSCs group, and the GO + UCMSCs group were higher than that in the blank group (*P* < 0.01), while the mean value of serum GAG in the GO + UCMSCs group was higher than that in the UCMSCs group (*P* < 0.05).

**Table 5:** Serum and articular fluid GAG results after treatment ( $\bar{x}$ ± s, ng/ml).

Group	Serum	Articular fluid	*P*
Blank group	18.709 ± 0.552	25.347 ± 0.561	0.010
GO group	22.689 ± 0.641^1^	30.123 ± 0.458^4^	0.015
UCMSCs group	24.028 ± 0.675^2^	30.719 ± 1.328^5^	0.046
GO + UCMSCs group	26.554 ± 0.450^3^	32.994 ± 1.347^6^	0.013

^1 – 6^Compared with the Blank group respectively, P values were 0.010, 0.004, and 0.000; 0.065, 0.022, and 0.009. P values for serum and articular fluid in the GO + UCMSC group compared to the UCMSC group were 0.011 and 0.245 respectively.

The mean value of articular fluid GAG in the GO + UCMSCs group was higher than that in the blank group (*P* < 0.01), whereas the mean value of articular fluid GAG in the UCMSCs group was higher than that in the blank group (*P* < 0.05). Besides, no statistically significant difference was observed between the GO group and the blank group (*P* > 0.05). Furthermore, when the GO + UCMSCs group and the UCMSCs group were compared, the difference in the mean was not statistically significant (*P* > 0.05). With respect to the results of GAG in serum and articular fluid, there were statistically significant differences in the blank group, the GO group, the UCMSCs group and the GO + UCMSCs group (*P* < 0.05).

The inflammatory cytokine levels demonstrated UCMSCs are more effective than GO granular lubricants in improving the inflammatory environment of the knee joint. In addition, the GO + UCMSCs group is the most effective in ameliorating the inflammatory internal environment of the knee joint. Nevertheless, the GO + UCMSCs group showed significant differences in inflammatory cytokine levels of serum and joint fluid, particularly in the levels of NO, IL-6 and TNF-α.

### Macroscopic Analysis

**Figure 8:**
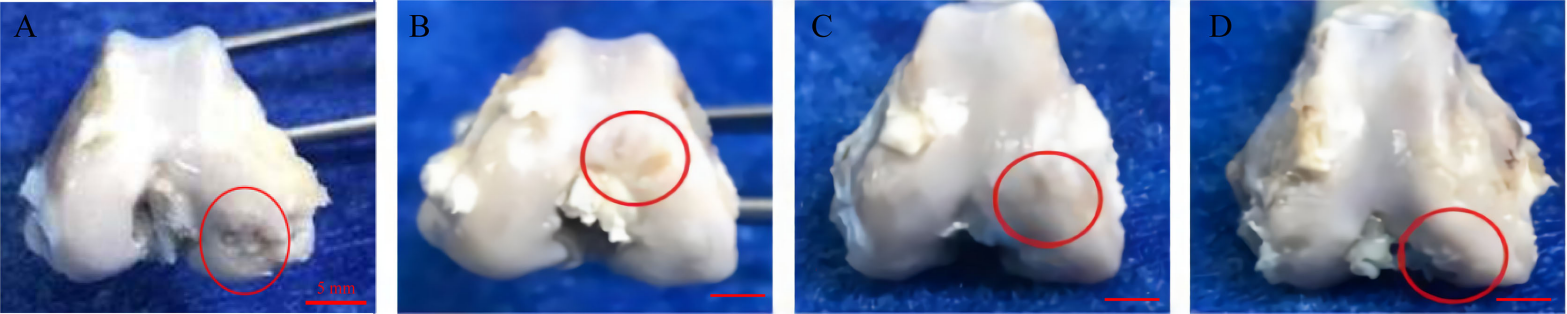
Representative macroscopic features of the femoral condyles. **(A)** blank group. **(B)** GO group. **(C)** UCMSCs group. **(D)** GO + UCMSCs group. Red circle: cartilage erosion area, scale bars = 5 mm.

The rabbits in the experimental group had no obvious synovial hyperplasia in the joint cavity, and there was a small amount of joint fluid. Besides, the areas of cartilage degeneration and defects were irregularly repaired in all treatment groups, and the repair was the most effective in the GO + UCMSCs group (**Figure 8**).

### Micro-CT Scan Results

**Figure 9:**
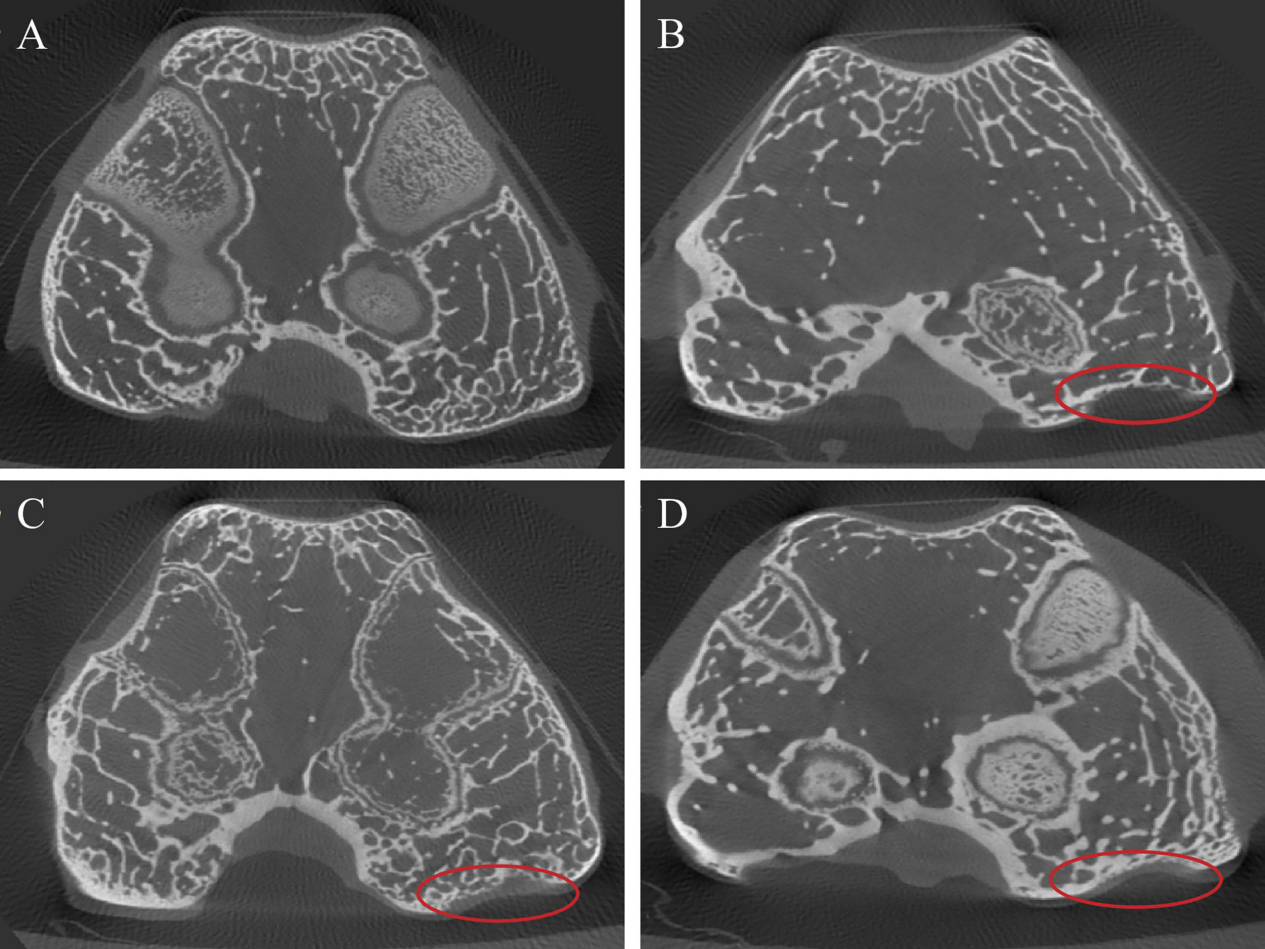
Micro-CT results of articular cartilage and subchondral bone after eight weeks of treatment. **(A)** blank group. **(B)** GO group. **(C)** UCMSCs group. **(D)** GO + UCMSCs group. Red circle: relatively normal area in OA joint.

The micro-CT results showed a certain degree of cartilage repair in each experimental group. In the GO + UCMSCs group, a smaller size cartilage defect could be seen on the medial condylar articular surface, smoothly connected to the surrounding cartilage tissue, with the bone quality of the defect close to normal cartilage and a shallow bone defect part, surrounded by a hypodense soft tissue shadow (**Figure 9D**). In the middle part of the medial condylar articular surface in the UCMSCs group, an obvious cartilage defect that accounted for about 1/3 of the cartilage on the medial condylar articular surface could be found. Apart from that, a small amount of continuous cartilage with uniform density, close to the normal cartilage tissue, was visible in the defect, and thin soft tissue was observed around it (**Figure 9C**). Moreover, in the middle part of the articular surface of the medial condyle in the GO group, an irregular cartilage defect could be seen, with a larger defect area, a deeper defect, and about 1/2 of the cartilage on the articular surface of the medial condyle, and an interrupted continuity (**Figure 9B**). Beyond that, in the blank group, the cartilage at the articular surface of the medial condyle was featured with uniform thickness, dense density and intact continuity, and besides, it was seen being surrounded by uniform and continuous hypodense shadow (**Figure 9A**). In sum, the images of the GO group, the UCMSCs group and the GO + UCMSCs group showed a progressive increase in the repair effect.

### HE Pathological Test Results

**Figure 10:**
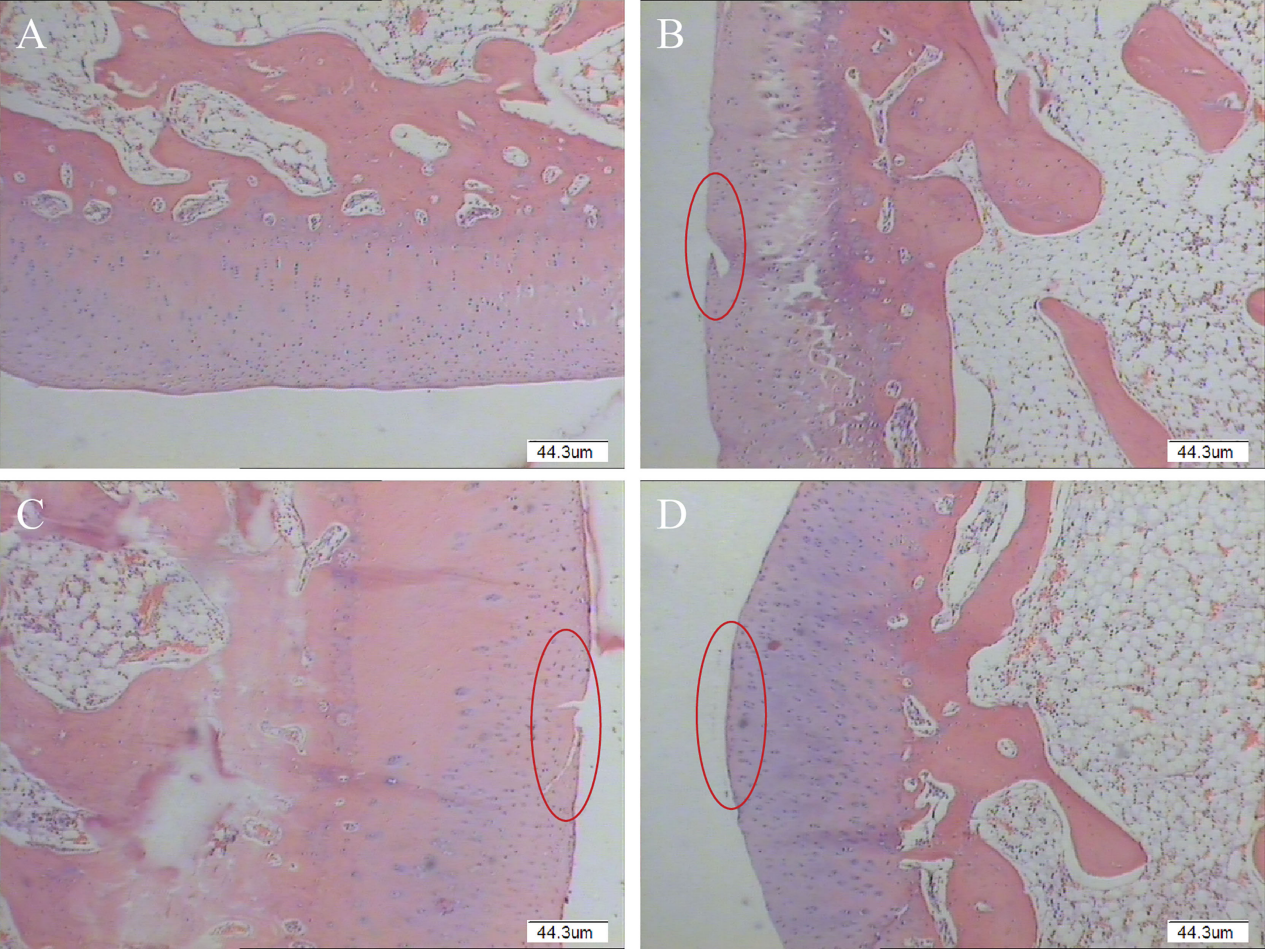
Pathological histological observation results of modified papain release joint injection group (Original magnification of histology images was × 40). **(A)** blank group. **(B)** GO group. **(C)** UCMSCs group. **(D)** GO + UCMSCs group. Red circle: cartilage erosion area, scale bars = 44.3 μm.

HE staining showed a smooth surface of cartilage in the GO + UCMSCs group, and the cartilage matrix was evenly colorized into light pink and blue chondrocyte nuclei. The cartilage exhibited clear-cut structural layers and a tidal line. The chondrocytes featured a certain stratification trend (**Figure 10D**). In the UCMSCs group, HE staining showed some new cartilage structure, but it was thinner than the original normal cartilage and had poor continuity with the subchondral bone, with fewer chondrocytes in the new structure and cracks on the cartilage surface (**Figure 10C**). Different from that, HE staining in the GO group displayed a large height difference compared to the normal cartilage layer, cracks on the rough cartilage surface, poorly layered chondrocyte structure, and no tidal lines (**Figure 10B**). Whereas, HE staining of the blank group was featured with the smooth cartilage surface, abundant chondrocytes, obvious layers and cell characteristics in each layer, and clear tidal lines (**Figure 10A**). In the joint injection group of modified papain to release agent, the cartilage repair effect of GO + UCMSCs, UCMSCs, and GO groups witnessed a gradient decrease, when that of the GO + UCMSCs group was the best. 

## Discussion

The pathogenesis of KOA is complex, with multiple cytokines and signaling pathways involved in the process. Cartilage degeneration occurs first, manifesting as progressive articular cartilage destruction and bone flab formation, accompanied by varying degrees of synovial inflammation (38–40). Articular cartilage is mainly composed of chondrocytes and the extracellular matrix, and the cytokines that regulate the function of KOA chondrocytes are mainly tumor necrosis factors, interleukins, and nitric oxide. Dysregulation of the relationship between synthesis and metabolism of these inflammatory cytokines can cause abnormal chondrocyte function, ultimately leading to cartilage matrix destruction and loss of joint function (41–44). Considering that, it has been revealed that NSAIDs and steroids are effective in the treatment of osteoarthritis, but their limitations, such as inability to prevent slowing cartilage degeneration and side effects, suggest that these drugs are not ideal for treating the disease (1, 45). Therefore, there is a growing interest in stem cell therapy for KOA, due to the enormous differentiation potential and immunomodulatory biological properties of stem cells (46–48). Besides, previous studies have found that GO particle lubrication has a reparative effect on the KOA model established in rats, and that low concentrations of GO particles are biocompatible with umbilical cord MSCs (34, 37), which promotes chondrogenic differentiation and osteogenic differentiation of human MSCs (35, 49–51). Hence, the anti-inflammatory effect of UCMSCs loaded with the GO particle lubricant on the papain-induced osteoarthritis model and the repair of articular cartilage and subchondral bone were evaluated *in vivo* in this study.

Inflammatory mechanisms play a key role in the pathophysiology of KOA (38, 40). The release of pro-inflammatory factors can contribute to the induction of NO synthesis by NO synthase, inhibit chondrocyte proliferation, and reduce the synthesis of the extracellular matrix. At the same time, NO can also directly damage the vascular wall, increase the permeability of the vascular wall and cause synovial congestion and edema and leukocyte exudation (52). TNF-α, as a common inflammatory mediator, can act alone or synergistically with other cytokines such as IL-1β and IL-6 to stimulate the production of cartilage, and synovial and subchondral bone layer-associated cells to produce matrix metalloproteinases, leading to gradual loss of cartilage collagen and proteoglycan and inhibition of proteoglycan and type II collagen synthesis, indirectly causing chondrocyte death and disrupting the homeostatic balance between cartilage damage and repair, which results in varying degrees of cartilage lysis and damage (53–55). Apart from that, IL -6 is capable of inducing chondrocyte IL-6 to participate in bone resorption by activating immature osteoclasts, while IL-6 stimulates chondrocytes and synoviocytes to produce prostate IL-6 and also participate in bone resorption by activating immature osteoclasts. Whereas, IL-6 stimulates the production of prostaglandins and collagenases by chondrocytes and synovial cells, leading to degeneration of articular cartilage (56–58). Furthermore, the expression level of pro-inflammatory cytokines is closely related to the severity of KOA disease (41, 42). In our project, intra-articular injection of papain was used to construct a knee osteoarthritis model, and the mechanism may be that papain hydrolyzes tissue in the knee joint cavity, and the necrotic products continue to produce an inflammatory response that causes damage to knee cartilage and surrounding soft tissues. Then, our data showed that umbilical cord MSCs reduced NO, IL-6, and TNF-α in serum and joint fluid and attenuated the inflammatory response in an animal model of knee osteoarthritis. UCMSCs also increased GAG and COL-II in serum and joint fluid, which are important cytokines secreted by chondrocytes and positively correlated with the degree of cartilage repair. The GO +UCMSCs group had the highest levels of GAG and COL-II expression, but the results of improved inflammatory factors in serum and joint fluid were more variable. Other than that, the level of factors in serum was also influenced by the overall condition of the model, whereas that in joint fluid mainly reflected the biochemical environment within the knee joint. In terms of overall trends, the results were consistent. Similar to the glycosaminoglycan and COL-II results, interleukin-6, tumor necrosis factor-α, and nitric oxide were reduced to the greatest extent in the GO + UCMSCs group, and it is possible that GO enhanced the anti-inflammatory effect of UCMSCs. Moreover, alterations in the pathological microenvironment of KOA are closely linked to the local release of multiple proteins and RNA translocations carried by exosomes, and among the 34 miRNAs associated with KOA, the MSC has the ability to inversely regulate the 34 miRNAs. As associated with KOA, in doing so, the repair process of intra-articular inflammation is mediated (59–61). Beyond that, MSC that possesses a good inflammatory repair effect and is able to converge towards the site of inflammatory response, can work precisely at the target site, and is featured with low immunogenicity; thus, it is suitable for inflammatory treatment of KOA (61, 62).

Subchondral bone remodeling is present throughout the pathogenesis of KOA, with enhanced bone resorption as the main pathological change in the early stages and subchondral bone sclerosis in the late stages (63, 64). Subchondral bone plays a key role in normal joint structure and function, providing nutrition for articular cartilage and influencing articular cartilage metabolism, in addition to its biomechanical support role (19). Considering that, inhibiting bone loss of subchondral bone, improving osteoporosis, and providing nutrition for articular cartilage can effectively slow down the degenerative damage of OA articular cartilage, which may be a potential target for KOA treatment (20). The results of the cartilage morphology and micro-CT scan in this study showed that the cartilage degeneration and defect areas were irregularly repaired in all treatment groups. To be specific, the cartilage and subchondral bone repair effects in the GO + UCMSCs, UCMSCs, GO and blank groups decreased in a gradient, when the GO + UCMSCs group was the best, with the thickest cartilage, more regular shape, closer contact with the surrounding cartilage, and less subchondral bone, which may be explained by the fact that biochemical and mechanical factors are important aspects of knee osteoarthritis pathogenesis. However, the umbilical cord MSCs were unable to produce better mechanical intervention on the articular cartilage surface. GO is a nanoscale granular material with good damping force characteristic and a smooth surface structure (32, 33), and it promotes osteogenic differentiation of stem cells and improves osteoporosis of the subchondral bone (35, 36, 51). The scaffold cell complex structure of GO-loaded MSC provides sufficient contact with the defective cartilage, including the surrounding normal cartilage, thus repairing the damaged cartilage, cartilage matrix, and subchondral bone. In contrast to recent approaches that focus solely on the therapeutic effects of MSC, the holistic effect of the scaffold-cell combination is more emphasized (49–51). The combination of the two has great advantages in improving the biomechanical environment in the joint cavity, and it can be seen as a composite structure of the joint fluid-like action in the closed joint cavity structure, carrying solid lubricating particles with a restorative effect on the cells that modulate the inflammatory action (65). Therefore, in this study, the degree of articular cartilage and subchondral bone repair was consistent with the trend in inflammation levels, suggesting that the GO + UCMSCs group is an effective treatment for KOA. Thus, it can be conclude that the combination of GO particulate lubricants with UCMSCs improved the therapeutic aspects of UCMSCs, reduced the level of inflammation in the internal environment of the knee joint, improved osteoporosis of the subchondral bone, and promoted articular cartilage repair.

As this study is still in the initial stages and has only demonstrated that GO granular lubricants are biocompatible with UCMSCs and therapeutic for KOA animal models, there are some limitations. Firstly, the effects of different concentrations of GO and HA on their intra-articular mechanical environment are different and need to be further investigated. Secondly, there is a lack of assessment of the cartilage mechanical environment before and after treatment that actually could be evaluated by SFA surface force instrumentation and atomic force microscopy to accurately analyze the treatment in each group. Finally, future studies should be conducted in depth in terms of matrix metabolism, the role of exosomes in UCMSCs. Moreover, a comprehensive evaluation of cartilage repair should be carried out by capturing the gait of the animal model in three dimensions. 

## Conclusion

UCMSCs loaded with the GO granular lubricants can promote chondrocyte secretion, reduce intra-articular inflammatory levels, ameliorate osteoporosis of the subchondral bone, and facilitate cartilage repair. 

## Data Availability Statement

The raw data supporting the conclusions of this article will be made available by the authors, without undue reservation.

## Ethics Statement

The animal study was reviewed and approved by the Animal experimental center at Tianjin University of Traditional Chinese Medicine.

## Author Contributions

AL participated in all experiments and was a major contributor in the writing of the manuscript. JZ and CZ provided theoretical guidance for experiments. AL was the main designer of the experiment. AL, JC, QZ, PN, and YY were responsible for the supply and detection of UCMSCs. YY was responsible for providing the GO granular lubricant. All authors read and approved the final manuscript.

## Funding

This work was supported by the National Natural Science Foundation of China (81873316).

## Conflict of Interest

The authors declare that the research was conducted in the absence of any commercial or financial relationships that could be construed as a potential conflict of interest.

## Publisher’s Note

All claims expressed in this article are solely those of the authors and do not necessarily represent those of their affiliated organizations, or those of the publisher, the editors and the reviewers. Any product that may be evaluated in this article, or claim that may be made by its manufacturer, is not guaranteed or endorsed by the publisher.
